# Hybrid Leadership for Māori Health: A Systematic Review

**DOI:** 10.3390/ijerph23050559

**Published:** 2026-04-26

**Authors:** Bridgette Masters-Awatere, Rachel McClintock, Utiku Potaka, Luke Enoka, Stacey Ruru, Amohia Boulton

**Affiliations:** 1 Māori and Psychology Research Unit, School of Psychology, University of Waikato, Private Bag 3105, Hamilton 3240, New Zealand; 2 Whakauae Research Services Ltd., 19 Ridgway Street, Whanganui 4500, New Zealandamohia@whakauae.co.nz (A.B.)

**Keywords:** Māori leadership, health leadership, hybridity, Te Tiriti o Waitangi, health equity, systematic review

## Abstract

**Highlights:**

**Public health relevance—How does this work relate to a public health issue?**

- This review links Māori leadership to the public health problem of persistent Māori health inequities, including how racism and system design shape access, quality, and outcomes in health services.
- It explains “hybrid leadership” as what Māori leaders must do in Crown-dominated health settings—holding accountability to iwi/communities while also meeting organisational, statutory, and professional requirements.

**Public health significance—Why is this work of significance to public health?**

- It argues that improving equity is not just a frontline issue—it is a governance and system issue—so strengthening Māori leadership is part of a core public health response.
- It shows that Māori leaders often carry heavy “translation” and relationship work without matching authority or resourcing, which can limit sustainable change and weaken leadership pipelines.

**Public health implications—What are the key implications or messages for practitioners, policy makers and/or researchers in public health?**

- For practitioners and policy-makers: do not rely on Māori leaders as “advisors” only—build clear Māori decision rights, resource Māori-led priority settings, embed tikanga in how decisions are made, and make equity/anti-racism accountable and measurable.
- For researchers: prioritise studies of Māori leadership in health governance, commissioning, and accountability, and investigate how leadership practices connect to equity outcomes (including the added barriers and burdens faced by wāhine Māori).

**Abstract:**

This systematic review synthesises the qualitative literature on Māori leadership to examine how leadership is conceptualised, enacted, and constrained, and what this implies for Aotearoa New Zealand’s health system. Across included studies, Māori leadership is grounded in whakapapa-based legitimacy, tikanga and mātauranga Māori, and collective responsibility for relational, cultural, and intergenerational wellbeing; these foundations persist across “traditional” and “contemporary” settings, with differences reflecting institutional conditions rather than shifts in core values. Interpreting the literature through a Māori cultural lens, the review shows that leadership is often exercised within Crown-dominated organisations where Māori authority is not the default, requiring leaders to navigate multiple accountabilities to iwi and communities, organisational mandates, and statutory obligations. Hybridity emerges as a structurally produced feature of practice, integrating Māori relational ethics with bureaucratic, professional, and governance requirements and ongoing translation work to make Māori priorities legible within institutional systems. Health-sector evidence illustrates how commissioning, funding, and accountability arrangements can limit Māori decision-making, increase leadership burden, and constrain sustainability and leadership pipelines. The review concludes that strengthening Māori leadership in health requires organisational and system change—such as clearer Māori decision rights, resourced Māori-led priority setting, and accountability mechanisms that operationalise equity and anti-racism—alongside targeted research on governance, commissioning, and system design.

## 1. Introduction

### 1.1. Māori Health Inequities and Te Tiriti Obligations in the Health System

Māori are the tāngata whenua (Indigenous people) of Aotearoa New Zealand. Māori society is organised through whakapapa (genealogy), with tribal structures comprising iwi (tribal nations) and hapū (sub-tribes) as the primary units of political, social, and cultural authority. Leadership, land, and collective identity are all grounded in whakapapa-based relationships that extend across generations and connect people to their rohe (ancestral territories). Contemporary Māori communities are diverse: Māori live across urban and rural settings, and many maintain active connections to iwi and hapū alongside participation in mainstream civic, professional, and institutional life. This diversity means that no single ethnographic profile captures the range of Māori realities; what is held in common is a shared Indigenous heritage, a set of cultural values and practices (tikanga) that ground collective life, and a political relationship with the Crown that is anchored in Te Tiriti o Waitangi of 1840.

Māori comprise a substantial and growing share of the population. Recent official health reporting places the Māori population at approximately 854,900 in 2020, with projections reaching around 1.1–1.2 million by 2040 [1,2]. These demographic realities matter because they sharpen, rather than dilute, the health system’s obligations to achieve equity and to engage with Māori in ways that reflect Māori needs and aspirations.

Across health scholarship and official inquiries, Māori health inequities are consistently treated as systemic and persistent, rather than as a residual problem of individual behaviour or “hard-to-reach” communities [3,4,5]. Evidence of inequitable access to, and experiences within, health care and related services is also apparent [6,7]. Racism is analysed in Aotearoa New Zealand health scholarship as a determinant operating across interpersonal, institutional, and structural levels, with implications for access, quality of care, and the distribution of health-promoting resources [5,7,8,9,10,11,12]. Consistent with this framing, inequity is positioned not only as a frontline workforce issue but also as a governance and system-design problem [5,9]. A further implication of this system framing is that workforce and leadership development are part of the equity response, not an optional add-on, particularly where Māori participation and progression are shaped by structural barriers [13].

These analyses heighten the salience of Te Tiriti o Waitangi in the contemporary health context, because Te Tiriti is repeatedly invoked in policy, legal, and inquiry settings as grounding Māori rights and the Crown’s duties in the design, funding, and accountability of public services [14,15,16]. Te Tiriti obligations are expressed as Māori participation in decision-making and accountability to Māori, and for equity rights when it comes to health access, outcomes and service experience [16]. This position is consistent with evidence that kaupapa Māori health interventions are often treated as both equity-oriented and rights-relevant, strengthening the argument for Māori-led approaches within system design [17].

### 1.2. Reforms and the Contemporary Governance Environment Māori Leaders Must Navigate

The current health system settings make leadership particularly consequential. The Pae Ora (Healthy Futures) Act 2022 came into force on 1 July 2022 and sets an explicit purpose of achieving equity, including by striving to eliminate health disparities, in particular for Māori (Pae Ora (Healthy Futures) Act 2022). This statutory framing sits alongside a longer period of critique and reform-oriented inquiry that characterised the health system as underperforming for Māori and as failing to give practical effect to Te Tiriti obligations in key areas of service design and delivery [3,16,18,19].

At the same time, the governance environment is not static. Te Aka Whai Ora, the Māori Health Authority established to advance hauora Māori and reduce inequities, was disestablished in 2024. Following legislative change, Manatū Hauora (Ministry of Health) and the Director-General of Health have assumed responsibility for functions previously held by Te Aka Whai Ora [20]. For Māori health leadership, this means leaders may need to sustain iwi (tribal) and community accountability while navigating shifting institutional mandates and decision-making rights [14,16]. Because this review includes literature published up to early March 2022, discussion of post-2022 reforms is included to situate interpretation and implications, rather than as evidence drawn from the included studies.

### 1.3. Why Contemporary Māori Health Leadership Implies Hybridity

Within this context, this article suggests that contemporary Māori health leadership is unlikely to be adequately captured by generic leadership competency lists alone. Leadership is better understood as practice within a contested interface, between iwi aspirations and accountabilities on one hand, and Crown statutory duties, public-sector rules, and organisational performance regimes on the other [14,15]. Māori leadership scholarship supports this practice-based framing, describing Māori leadership as enacted within institutional constraints and within ongoing Māori responsibilities to community, culture, and kaupapa [21,22,23].

Complementary arguments from Māori leadership scholarship in the public sector characterise Māori leadership practice as involving strategic positioning to advance kaupapa Māori (agenda) within Crown spaces, alongside deliberate navigation of bureaucratic pressures associated with dominant governance norms [21]. In Māori public health leadership research, leadership is similarly described as requiring capability to operate across cultural and institutional contexts, including working collectively and negotiating “two worlds” demands [23]. Taken together, these sources support treating hybridity as an analytically useful framing: leaders are often required to hold Māori relational and collective accountabilities while engaging fluently with formal public-sector duties, regulatory frameworks, and institutional constraints [21,23]. This argument is further strengthened by contemporary Māori leadership theory, which explicitly conceptualises collective and relational leadership as a distinct paradigm rather than merely a cultural overlay on Western leadership models [24,25,26].

Hybridity does not require an assumption that Māori priorities should be diluted to fit existing systems. Rather, it foregrounds the capability demands placed on Māori health leaders when they are expected to translate, negotiate, and sometimes redesign institutional settings so that Māori authority, tikanga (customs) processes, and equity objectives can be given operational effect [14,15,16]. Māori leadership scholarship also suggests that leadership identity and legitimacy can draw on collective memory and cultural continuity, which helps interpret why Māori leaders may treat the protection of tikanga and relationships as integral rather than discretionary when operating in institutional settings [26,27].

Against this backdrop, this review has three aims: first, to synthesise qualitative literature on Māori leadership in order to identify foundational principles and how they are enacted across institutional contexts; second, to examine how hybridity functions as a structural condition of Māori health leadership, rather than as a style choice; and third, to draw out implications for health system practice, policy, and future research. The review is motivated by the persistent gap between Māori health equity obligations and system performance, and by evidence that leadership development alone is insufficient without corresponding change to the institutional conditions in which Māori leaders work. It is intended to be of direct relevance to health-sector practitioners, commissioners, governance bodies, and researchers working to give practical effect to Te Tiriti obligations and health equity commitments.

## 2. Materials and Methods

We undertook a systematic literature review and qualitative meta-synthesis to identify and interpret Māori perspectives on leadership, with the aim of understanding how they can contribute to leadership in health. The review was guided by an overarching Kaupapa Māori philosophical orientation and was reported in line with PRISMA 2020 for systematic reviews and the ENTREQ guidance for transparent reporting of qualitative syntheses [28,29,30]. A tuakana-teina (older-younger sibling) approach to the systematic review ensured that learning occurred in both directions, with senior members of the team guiding the work undertaken by an emerging researcher, and that researcher in turn teaching the senior researchers’ new tools and techniques to facilitate the review. Team members were located in a number of different regions, so regular online wānanga (deliberative forum) to discuss the methods, process, and results with all team members were critical to the integrity of our kaupapa Māori approach. In keeping with systematic review principles, our approach prioritised transparency and replicability in searching, screening, and synthesis [31,32]. Due to the tight timeframe to conduct this research, the review was not registered. Electronic searches were conducted in late February and early March 2022 across 12 bibliographic databases and two dedicated websites relevant to Aotearoa New Zealand and Kaupapa Māori scholarship (see Supplementary Table S1, Supplementary S2, Supplementary S3 and Supplementary S4). In addition, the reference lists of all included full-text articles were hand-searched to identify any additional eligible studies.

Note on terminology and navigation: Māori language terms are used throughout this manuscript. Definitions for all Māori terms are provided in the Glossary at the end of the article. Information sources searched are listed in Table S1; the rationale for each source is provided in Supplementary S2. Full search strings, database limits, and result counts are provided in Supplementary S3, Supplementary S4 and Supplementary Table S5. The PRISMA 2020 checklist and ENTREQ statement are provided in Supplementary Table S6 and Supplementary Table S7, respectively.

Because Māori scholarship can be disseminated through a range of formats, we included both peer-reviewed literature and relevant grey literature, provided that full texts were retrievable. Charting of grey literature reduced the risk of systematically excluding Indigenous research and knowledge that may not be represented in conventional journal publishing pathways [33,34]. Google Scholar was searched to support capture of both formal and grey literature, recognising its breadth alongside limitations in reproducibility and ranking effects [35,36]. For searches yielding very large result sets in Google Scholar and ProQuest, only the first 200 results per search string were imported for screening, consistent with established guidance for managing volume while retaining relevance [35].

### 2.1. Positionality

This work is undertaken as part of a project entitled (name removed to preserve double-blind review) which sits within a larger, five-year programme of research. All of the authors, members of the project team, are Māori. Collectively the team have overlapping and extensive experience in health, research, policy, governance and tribal leadership. The project benefits from cultural oversight provided by a Tikanga Advisory Group established for the programme.

### 2.2. Eligibility Criteria

Inclusion criteria required qualitative empirical research that prioritised primary data and was published in English. Included texts needed to foreground Māori perspectives on health leadership, including leadership models, cultural values, skills, abilities, and processes described as relevant to Māori leaders (both traditional and contemporary). “Health” was treated holistically, extending beyond biomedical framings and the boundaries of the formally funded health system to include socio-cultural, socio-political, and intergenerational dimensions connected with whakapapa (cultural identity), whenua (land) and taiao (environment). Because the review aimed to inform iwi Māori perspectives of health leadership, Māori leadership literature situated in iwi contexts was also eligible. To privilege Māori voices, we included studies in which all or most participants were Māori and in which Māori perspectives were central to the analysis. We preferentially included literature authored by Māori research teams or with Māori lead authorship. If at least one inclusion criterion was not met, a study was excluded. Publications written in te reo were not included. If there was any ambiguity about inclusion criteria being met, a study moved to the next round of review.

Health texts were limited to those published from January 2000 to early March 2022. This date range was selected to align the review with major structural changes in the Aotearoa New Zealand health sector during this period, while retaining sufficient depth to capture the evolution of Māori leadership discussions across policy and service contexts. Exclusion criteria were applied in three areas: (1) reflexive or conceptual pieces that did not present new empirical findings on Māori health leadership relative to their publication timing; (2) texts focused on whānau (family) or patient-level decision-making rather than leadership decision-making; and (3) texts where Māori leadership was only a minor component rather than a substantive focus.

### 2.3. Screening and Selection

**Figure 1:**
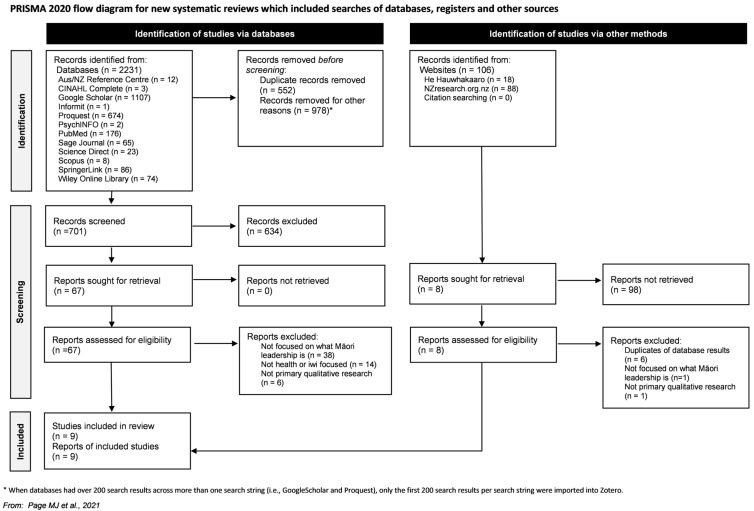
PRISMA 2020 flow diagram [28].

Our process followed Bramer and colleagues’ recommended [37] approach and ensured consistency with documentation details [28]. Search results were imported into Zotero, where duplicates were identified and merged. Screening proceeded in two rounds (summarised in the PRISMA flow diagram presented in Figure 1). In round one, titles, abstracts, and available summaries were screened against eligibility criteria. Where abstracts were unclear, items progressed to full-text review, to avoid premature exclusion. A concept matrix was then developed to track the presence of key concepts across shortlisted items and to support consistent decision-making. The concept matrix template and decision rules are reported in Supplementary S8 and Table S9. In round two, full texts were retrieved and reviewed independently by multiple team members, then discussed collectively to reach agreement on inclusion and to document reasons for exclusion.

### 2.4. Appraisal and Data Extraction

Recognising ongoing debate about how to appraise qualitative research, particularly when Indigenous scholarship spans varied publication types and epistemic foundations, we used a reflexive dialogue approach during full-text review, consistent with qualitative synthesis guidance that emphasises transparency and reflexivity over rigid scoring [29,38,39]. Because of the appraisal approach used, the literature was not given a score; instead, discussions by the research team determined whether to include/exclude sources. After discussing the shortlisted items, none were excluded. As texts were confirmed for inclusion, key details were extracted into a charting table, including author and year, aims, location, participants, methodological framing, data collection, analytic approach, and publication type (see Table S10).

### 2.5. Analysis and Synthesis

We used an inductive thematic analytic approach to synthesise findings across included texts. First, relevant excerpts were identified from results and discussion sections before being descriptively coded in Zotero (version 5) [Computer software] Corporation for Digital Scholarship www.zotero.org (accessed on 8 September 2021) was used to store and manage the coding process. Codes were iteratively refined and organised, then grouped into higher-order categories representing patterns across the literature. Coding and excerpt management were supported using qualitative analysis software. In the second stage, coded excerpts were re-read and summarised into narrative syntheses within each category, which then informed the structure and content of the findings and discussion [39]. The software Dedoose (version 9.0.17)—Computer software. SocioCultural Research Consultants. www.dedoose.com (accessed on 5 November 2021) was used to assist with coding and filing of data.

### 2.6. Limitations

A limitation of this review arises from the distribution and focus of the available Māori leadership literature. While there is a substantial and theoretically rich body of work examining Māori leadership attributes, values, and practices, much of this literature is grounded in cultural, iwi, public sector, education, and business contexts, with comparatively limited empirical attention to leadership within health service governance, commissioning, and organisational decision-making settings [21,22,23,25]. As a result, the analysis necessarily draws on leadership scholarship developed outside the health sector to interpret how Māori leadership principles are enacted within health contexts, requiring an interpretive step in which concepts such as hybridity, translation labour, and dual accountability—well articulated across Māori leadership research—are applied to health settings where direct empirical evidence remains uneven. At the same time, this cross-sector synthesis strengthens theory-building by enabling identification of enduring leadership principles, what we have termed foundational principles (whakapapa-based legitimacy, tikanga and mātauranga Māori, collective responsibility, and relational ethics), that persist across contexts, rather than treating health leadership as an isolated domain. By tracing how these principles are translated within highly regulated institutional systems, the review positions hybridity as a structural feature of Māori leadership in Crown-dominated environments, including health. In this sense, the review contributes a coherent conceptual bridge between Māori leadership theory and health system practice, while also highlighting a clear need for future empirical research focused specifically on Māori leadership within health governance, commissioning, and accountability settings.

### 2.7. Use of Generative Artificial Intelligence (GenAI)

ChatGPT-5.2 was used as a language-support tool to draft summaries, improve narrative consistency, and moderate tone towards a cautious, evidence-based style. All AI-assisted text was subsequently reviewed, validated, and edited by the authors.

## 3. Findings

### 3.1. Foundational Principles of Māori Leadership

Across the included literature, Māori leadership has consistently been described as a single, evolving leadership tradition in which foundational principles (whakapapa, tikanga, collective responsibility, relational ethics) are continuously enacted, translated, and negotiated within changing institutional contexts. The distinction between “traditional” and “contemporary” leadership is therefore primarily analytic rather than substantive: the same core principles underpin leadership across time, while the conditions of enactment—particularly within Crown and health-sector institutions—require increasingly hybrid forms of practice [21,22,23,40,41].

The sections that follow deliberately build toward the hybrid leadership analysis. Four foundational principles of Māori leadership are outlined to facilitate understanding of our proposed hybrid leadership, which in this context is Māori leadership in Western institutional environments such as the health sector.

#### 3.1.1. Whakapapa-Based Leadership

Whakapapa-based leadership legitimacy is closely tied to sustaining relationships and meeting collective responsibilities over time, extending evaluation beyond immediate organisational goals [25]. The literature distinguishes between whakapapa-based and kaupapa-based collectives, noting that leadership may be organised through genealogy, kaupapa, or combinations of both depending on context [23,42,43]. This flexibility is important for understanding leadership in health and public sector settings, where kaupapa-based collectives are common but whakapapa-based accountabilities persist.

Leadership development is closely linked to intergenerational transmission of knowledge, including whakapapa, pūrākau (narratives), whakataukī (proverbs), and toi (art) forms, which function as governance and ethical resources rather than cultural embellishments [26,41]. At the same time, colonisation and Western institutional dominance are described as disrupting Māori knowledge transmission and producing uneven access to cultural resources, shaping who is able to enact leadership and under what conditions [21,22,23,44]. This uneven access helps explain why contemporary Māori leadership frequently requires translation and protection of tikanga; an issue that becomes central in hybrid leadership analysis.

#### 3.1.2. Tikanga and Mātauranga Māori

Leadership across the literature is consistently framed as enacted practice grounded in tikanga and mātauranga Māori, rather than as an abstract or transferable competency set [25,40,41]. Leadership is made visible and accountable through practice, particularly in collective settings such as hui (gatherings), pōwhiri (welcome ceremony), and tangihanga (funeral ceremony), where leaders are evaluated through their conduct, cultural competence, and relational integrity [40,41,42].

This practice-based understanding is critical for interpreting hybrid leadership later in the findings. Because leadership legitimacy is enacted publicly and relationally, Māori leaders carry tikanga-based responsibilities with them when operating in institutional environments where tikanga is not structurally embedded [21,23,45].

Contemporary Māori leadership literature explicitly extends tikanga-based practice into public sector, health, education, and organisational settings, where leaders are expected to uphold mana, tikanga, and cultural integrity alongside professional and statutory obligations [23,42]. Cultural capability, including te reo Māori and tikanga knowledge, is therefore treated as integral to leadership legitimacy, not as an optional or symbolic attribute [45].

#### 3.1.3. Collective Responsibility for Wellbeing

A third foundational principle, collective responsibility, connects leadership purpose directly to wellbeing, including health and intergenerational outcomes. Across the literature, Māori leadership is consistently associated with manaakitanga (hospitality), whanaungatanga (relationship), and kaitiakitanga (stewardship), framing leadership as responsibility for people, relationships, whenua, and future generations [22,25,40,41]. These cultural values inform both leadership purpose and evaluation, extending accountability beyond immediate organisational goals to collective and intergenerational wellbeing [25].

The literature distinguishes between whakapapa-based and kaupapa-based collectives, noting that leadership may be organised through genealogy, kaupapa, or both depending on context [23,42,43]. They can also include non-Māori [21,23,46]. Representation and accountability to the collective contributes to feelings of belonging and responsibility [40,42,46]. These complexities reinforce collective responsibility as a core principle, rather than a fixed institutional form.

#### 3.1.4. Relational Ethics

Māori leadership is repeatedly described as value-saturated rather than value-neutral, with ethical commitments inseparable from leadership practice [22,25,45]. These ethical commitments directly shape how Māori leaders interpret their responsibilities in institutional roles, particularly where organisational performance imperatives conflict with Māori responsibilities for collective wellbeing. This ethical orientation creates the conditions under which hybrid leadership becomes necessary rather than optional.

In responding to the dynamic shifts in sociopolitical and economic climates, future Māori health leadership capability development must necessarily prepare leaders for (a) Te Tiriti-literate governance practice across iwi–Crown interfaces, (b) commissioning and accountability settings that can shift through legislative and organisational change, and (c) organisational change work where racism and inequity are treated as system-level problems requiring measurable institutional response [5,7,9,16]. In addition, evidence on kaupapa Māori interventions supports preparing leaders to interpret and steward Māori-led models of care within commissioning and accountability environments, including how “evidence” is framed and legitimised in policy and service design [17].

### 3.2. Hybrid Leadership in the Health Sector

Hybrid leadership emerges logically from the foundational principles of Māori leadership outlined above, rather than as a distinct or optional leadership style. Because Māori leadership is grounded in whakapapa-based legitimacy, enacted through tikanga, oriented toward collective wellbeing, and concerned with the maintenance of ethical relationships Māori leaders operating in contemporary institutional environments must integrate these responsibilities with organisational systems where Māori authority is not the default [21,23]. Hybridity therefore reflects the practical conditions under which Māori leadership is exercised, rather than a departure from foundational principles.

Across the literature, Māori leadership is described as requiring fluency across Te Ao Māori and Western organisational worlds, particularly in public sector and health contexts [21,23]. This fluency is not framed simply as bicultural competence or interpersonal skill. Instead, it is described as a structural condition of leadership in Aotearoa, shaped by colonisation, Treaty relationships, and the persistent marginalisation of Māori authority within dominant governance frameworks [24]. Māori leaders are therefore positioned at the interface of multiple systems of accountability, simultaneously responsible to iwi and Māori communities, and to organisational mandates, statutory duties, and professional norms [21,23].

Hybrid leadership involves the active integration of Māori relational ethics, manaakitanga, whanaungatanga, and responsibilities consistent with rangatiratanga, along with institutional expectations such as compliance, performance management, and bureaucratic accountability [25,45]. Leaders are not described as alternating between “Māori” and “Western” leadership modes, but as holding these demands together contemporaneously in practice, often under conditions of constraint. The need to balance these value-bases simultaneously reinforces earlier findings that Māori leadership is value-saturated rather than value-neutral, and that ethical commitments cannot be suspended when leaders enter institutional roles [25,26,45].

A defining feature of hybrid leadership across the literature is the notion of “translation labour”. Māori leaders are described as continually translating Māori priorities, values, and relational obligations into institutional language and processes that are legible within Western governance systems [21,23]. Such activity includes reframing kaupapa Māori goals into strategic plans, performance indicators, or funding justifications; creating space for tikanga within organisational routines; and protecting Māori integrity where dominant systems marginalise mātauranga Māori [21]. This translation work is not episodic, but ongoing, reflecting the earlier finding that leadership legitimacy is enacted and relational rather than fixed.

Translation labour is distinct from bicultural competence or cultural awareness. It is not a personal skill set that individual leaders can acquire through training; it is a structural demand produced by the misalignment between Māori leadership values and the design of Crown-dominated institutions. Because institutional systems are not built around Māori authority, Māori leaders must continuously convert, explain, and reframe their priorities in order to participate in processes that were not designed with those priorities in mind. This work is cumulative and distributed unevenly: leaders in more isolated positions, with less peer support or organisational backing, carry a disproportionate share of it. The consequences for leadership sustainability are addressed in Section 3.2.2.

Hybrid leadership is therefore not simply the application of “traditional values” in modern contexts. Instead, it involves ongoing negotiation, mediation, and ethical judgement, as leaders seek to advance Māori priorities within institutional environments shaped by non-Māori assumptions about authority, evidence, and accountability [24,25]. Thus, Māori leadership can be viewed as a distinct relational and collective paradigm, rather than as a cultural overlay on Western leadership models [25,45].

#### 3.2.1. Enactment of Hybrid Leadership

The manner in which the health system combines high levels of institutional regulation with strong expectations that Māori leaders contribute to equity, cultural safety, and Treaty responsiveness [23] exemplifies hybrid leadership in practice. In public health contexts, Māori leadership is described through patterns such as “walking in two worlds” and “working as a collective,” capturing the need to balance Māori relational obligations with bureaucratic, biomedical, and Crown-derived governance frameworks [23].

Māori public health leaders are often positioned as intermediaries between Māori communities and state institutions. They are described as advancing kaupapa Māori and collective wellbeing goals while simultaneously navigating funding models, professional standards, reporting requirements, and organisational performance expectations [21,23,47]. Such positioning reflects the earlier finding that Māori leadership accountability frequently extends beyond organisational boundaries, encompassing obligations to whānau, hapū, iwi, and Māori communities, even when leaders hold formal institutional roles.

Leadership in health services and governance settings is frequently enacted in environments where Māori participation is present, but authority is constrained. Māori leaders are described as contributing to service leadership, advisory groups, and commissioning processes without commensurate decision-making power over resourcing or system design [21,23]. Consequently, structural misalignment between responsibility and authority can occur where Māori leaders are accountable for relationships, cultural integrity, and equity outcomes, but lack control over the levers required to effect systemic change.

Health-sector evidence situates these dynamics within broader patterns of institutional racism and inequitable governance processes, reinforcing that leadership challenges are system-level rather than individual [7]. Māori leaders are frequently expected to provide cultural expertise, equity insight, and relational labour to compensate for system deficiencies, intensifying leadership burden without addressing underlying power imbalances [21,23].

Hybrid leadership in health is also shaped by the instability and complexity of governance environments. Leaders are described as needing to maintain accountability to Māori communities while navigating shifting organisational mandates and policy settings, reinforcing the importance of adaptability alongside cultural grounding [21,23]. Navigating accountability across multiple dynamics further illustrates how hybrid leadership arises from foundational Māori leadership principles meeting institutional volatility, rather than from individual preference.

The health sector imposes specific conditions that intensify the demands of hybrid leadership beyond what is typical in other public-sector or business contexts. First, health services operate under high levels of statutory, clinical, and professional regulation, meaning Māori leaders must integrate tikanga and Kaupapa Māori priorities within accountability frameworks that carry legal and professional consequences. Second, leadership decisions in health have direct and measurable consequences for Māori health outcomes, making the stakes of inadequate institutional design particularly concrete for access, quality of care, and health equity. Third, the health evidence base documents racism operating at structural, institutional, and interpersonal levels [7,8,9,10,11], which means that addressing institutional racism is not merely a values aspiration for Māori health leaders, but a system-level challenge embedded in their daily practice. Fourth, in commissioning and contracting environments, Māori leaders must protect kaupapa Māori models of care within funding systems that may not recognise Māori-defined evidence or community-defined outcomes [17,47]. Together, these conditions mean that the health sector is not simply one context among others for hybrid leadership; it is one of the most structurally demanding.

#### 3.2.2. Constraint, Burden, and Sustainability

Across sectors, hybrid leadership is portrayed as adaptive and ethically demanding, but also fragile where institutional conditions remain unchanged. The literature identifies constraints and pressures that are associated with leading in systems shaped by colonisation and power asymmetries [21,23,41]. In public sector and public health contexts, Māori leaders are described as navigating dominant governance models and bureaucratic norms that can marginalise mātauranga Māori and kaupapa Māori priorities [21,23]. This marginalisation is situated within broader accounts of inequities, power imbalances, under-representation of Māori in leadership, and limited recognition of Māori approaches in institutional settings [21,23,41,42,44,48]. These constraints shape not only leadership practice but also leadership sustainability.

Gendered barriers are also reported, with Māori women leaders describing discrimination and constrained leadership pathways, including compounded effects where racism and sexism intersect [41,48,49]. The literature further suggests that Māori leaders are frequently expected to contribute to wider social change, challenge stereotypes, and support Māori language and culture, which can intensify leadership burden in institutional settings [22,23,45,49]. These pressures are not framed only as interpersonal costs; they are also linked to structural conditions, including cultural representation expectations and decision-making arrangements where authority may not sit with Māori [21,23]. Evidence from wider health research amply demonstrates the links between racism, institutional processes, and measurable inequities, indicating the importance of system-level responses rather than individualised solutions [7].

The barriers facing wāhine Māori in leadership require a more specific account. The included literature documents that wāhine Māori navigate compounded structural disadvantage arising from the intersection of colonisation, racism, and sexism—conditions that are not simply additive but mutually reinforcing [41,48,49]. This analysis is grounded in mana wāhine theory, a distinctly Māori epistemological framework that centres the authority, dignity, and power of Māori women [50,51,52]. Mana wāhine is not a local adaptation of Western intersectionality theory; it theorises the position of wāhine Māori through te ao Māori concepts of mana, whakapapa, and tikanga, including recognition that pre-colonial Māori society held distinct and complementary roles for men and women, and that colonisation introduced patriarchal structures that eroded the authority and leadership standing of wāhine Māori [53,54,55]. In the health leadership context, this means that wāhine Māori leaders may face institutional environments shaped not only by Crown governance norms but by colonially imposed gender hierarchies that have no basis in tikanga. Leadership development and governance design must therefore address these structural conditions explicitly, rather than treating wāhine Māori leadership barriers as incidental to the broader equity agenda.

Across the literature, Māori leaders are frequently expected to contribute to broader social change, challenge inequities, and support cultural revitalisation, adding to the leadership burden already experienced in institutional settings [22,23,45]. Importantly, these pressures are not framed as individual shortcomings. Instead, they are linked to structural conditions in which Māori leadership is relied upon to adapt to systems that have not been designed to share authority or redistribute power [21,23]. Without institutional change, hybrid leadership risks becoming a mechanism for sustaining inequitable systems, with Māori leaders absorbing the costs of translation, mediation, and relationship maintenance.

Taken together, the hybrid leadership literature reinforces the earlier foundational findings: because Māori leadership is relational, ethical, and collective, Māori leaders cannot disengage from responsibilities when institutional environments are misaligned. Hybrid leadership therefore represents both the resilience of Māori leadership traditions and the limits of leadership in the absence of structural transformation, particularly within the health sector.

## 4. Discussion

This synthesis has direct relevance for Māori leadership of health services because the literature does not depict Māori leadership as a transferable “style” that can be inserted into any organisational setting without changing conditions of authority and accountability [21,23]. Māori leadership is described as relational, collective, and responsibility-based, while contemporary leadership is frequently enacted within institutions shaped by non-Māori norms and power structures [21,23,25,45]. In this context, leadership development is unlikely to be sufficient on its own, because the viability of Māori leadership practice depends on the institutional settings in which Māori leaders operate [21,23]. The Treaty-based context is particularly important for health governance, where organisational interpretations of Te Tiriti obligations shape Māori authority, participation, and accountability in practice [15].

The question of whether hybrid leadership is workable in practice deserves direct attention. The review does not claim that the tension between Māori collective and relational values and Crown linear governance structures is costless or easily resolved. Rather, it argues that this tension is the structural reality within which Māori health leaders already operate, and that the relevant question is what conditions make practice more or less sustainable. Evidence from the included literature indicates that workable hybrid practice does exist. Bean [21] documents Māori public sector leaders successfully advancing kaupapa Māori priorities within Crown governance frameworks through deliberate collective leadership structures and strategic positioning. Tipene [23] describes Māori public health practitioners maintaining cultural accountability while navigating bureaucratic environments, sustained by wānanga and peer network structures. Boulton [47,56] provides health-sector evidence that kaupapa Māori mental health providers adapted Crown contracting processes in ways that protected tikanga-based service design. These examples show that hybrid leadership is not inherently inviable; what makes it fragile is not the values tension itself but the absence of institutional conditions that reduce the burden on individual leaders to manage that tension alone. Where Māori leaders lack decision rights, resourcing, or protected space for tikanga-based practice, the workability of hybrid leadership diminishes. The review’s implications are therefore directed at those institutional conditions rather than at individual capability.

A related question is whether the form of Māori leadership described in this review can only be fully expressed within Māori-governed or kaupapa Māori services. The evidence from Boulton [47,56] does indicate that services governed and delivered by Māori provide conditions most naturally aligned with relational ethics, tikanga-based practice, and accountability to community, and in that sense reduce the structural misalignment that characterises hybrid leadership elsewhere. However, the review’s argument is not that Māori leadership is only viable in Māori-controlled settings. Most Māori health leaders work within mainstream and Crown-dominated organisations, and the review is primarily concerned with strengthening conditions in those settings. The implication is that mainstream organisations need to actively redesign governance, commissioning, and accountability structures so that Māori leadership can be exercised with integrity—not that Māori leadership should be confined to kaupapa Māori services, though those services offer important design principles for the mainstream.

Health services seeking to strengthen Māori leadership need to recognise that leadership legitimacy is tied to obligations beyond the organisation itself, including responsibilities to whānau, hapū, iwi, and Māori communities [41,44]. Leadership decisions can affect mana, wellbeing, and intergenerational continuity, which can generate ethical tensions where organisational performance imperatives conflict with Māori responsibilities [23,45]. Under these conditions, Māori leadership roles require not only cultural competence but also clear authority, resourcing, and decision rights to act consistently with Māori priorities, rather than being confined to advisory or symbolic roles [16,57,58].

Evidence of “two worlds” practice indicates that Māori leaders in health contexts may need to perform substantial translational labour, including navigating institutional settings while maintaining Māori ethical commitments and collective responsibilities [21,23]. The navigation required of Māori leaders is already positioned within a hostile environment combining colonisation, power imbalances, and the under-recognition of Māori approaches, which can intensify the burden placed on Māori leaders to enable change within systems that have not been designed for Māori authority [21,23,48]. Although overload and attrition are not consistently measured outcomes in the Māori leadership studies cited, the combination of sustained structural pressures and expectations that Māori leaders carry cultural and social change responsibilities, makes the risk of overload a plausible concern for workforce sustainability in health services [21,23,45,49].

Leadership approaches that distribute leadership work collectively rather than relying on individualised leadership models are highly desirable from a Māori perspective. Collective leadership structures, strong Māori peer networks, and organisational accountability mechanisms are consistent with evidence that Māori leadership is commonly enacted in collective, relational ways. Furthermore, evidence shows that organisational and iwi support are critical for leadership development and enactment [21,23,25,40,42,44].

Intersectional barriers also require explicit attention. The literature on wāhine Māori leadership reports discrimination and constrained leadership pathways shaped by both sexism and racism, indicating that health services should not assume leadership pathways are neutral. Rather, health services must look to actively address structural barriers in the recruitment, development, and promotion of Māori leaders [41,48,49].

The structural barriers facing wāhine Māori in health leadership pathways require explicit and sustained attention. As discussed in Section 3.2.2, these barriers arise from the compounded effects of colonisation, institutional racism, and gender discrimination—conditions analysed through mana wāhine theory as producing multiple intersecting sites of disadvantage [50,51]. Health services cannot treat these barriers as peripheral to equity work. Recruitment, development, and governance processes should be examined for structural bias, and active measures should be taken to protect and resource wāhine Māori leadership pathways. This requires going beyond cultural competence training to address the organisational power dynamics and decision-making arrangements that have historically excluded or marginalised wāhine Māori from leadership positions commensurate with their knowledge, authority, and community accountability.

Finally, the literature suggests that leadership practice is closely connected to tikanga and mātauranga Māori as governance and ethical systems, rather than as cultural additions to existing organisational routines [25,36,40,45,59]. For health services, this implies that tikanga-based practice should be embedded in leadership and service design, including how decisions are made, how accountability is expressed, and how relationships with Māori communities are sustained [15]. Achieving this is likely to require organisational learning and power-sharing, rather than expecting Māori leaders to adapt Māori leadership into unchanged institutional defaults [21,23,57].

### 4.1. Implications for Future Research

The presence of a strong literature base provides rich, context-specific accounts of Māori leadership; however, demonstration is uneven across settings. An absence of Māori leadership literature was particularly noted for health service governance and management contexts where decisions about resourcing, commissioning, and system design are made [21,23]. Greater empirical attention to Māori leadership within health services is warranted, including leadership roles across kaupapa Māori providers, mainstream providers, commissioning and governance bodies, and clinical leadership contexts, given ongoing concern in the health sector about Te Tiriti implementation and Māori representation in decision-making [15,16,57,58].

Future research also needs to strengthen explanations of how leadership practice links to better outcomes. While the current literature emphasises relational ethics and collective orientation, there is less direct evidence specifying mechanisms through which Māori leadership influences equity-relevant outcomes within health services, such as improved access, patient-defined cultural safety, or organisational accountability for equity [25,45]. Studies that examine these pathways can be strengthened by Indigenous methodologies and Kaupapa Māori approaches that centre Māori priorities, knowledge systems, and community accountability [60,61].

Comparative research is also important, provided it avoids pan-tribal generalisation. The literature explicitly cautions that Māori leadership varies between iwi and hapū and is enacted differently by sector and context, suggesting value in comparative studies that remain tikanga grounded and locally accountable while identifying shared principles and divergent enactments [23,42,43]. In addition, research on leadership trajectories for wāhine Māori should be expanded, including how racism and sexism operate together in health leadership pathways and what organisational interventions most effectively remove barriers [7,41,48,49].

Intergenerational dimensions of leadership represent a further evidence gap. The included literature addresses intergenerational leadership through whakapapa-based succession and tuakana-teina knowledge transmission (Section 3.1.1), which are the conceptual categories most appropriate to te ao Māori understandings of seniority and leadership development. However, there is limited empirical research specifically examining how leadership trajectories unfold across the career span for Māori health leaders, or how age-related factors (including access to kaumātua knowledge and the structural barriers facing rangatahi (younger) leaders interact with institutional conditions. Future research in this area should be grounded in Māori conceptual categories rather than importing Western career-stage frameworks that may not reflect the relational and whakapapa-based nature of Māori leadership development.

### 4.2. Implications for Practice and Policy

The literature supports practice and policy responses that address leadership development, organisational design, and system accountability. Because Māori leadership is frequently described as relational and collective, leadership training for Māori in health services should reflect collective capability building and relational accountability, rather than focusing solely on individual competencies [23,25]. Mentoring and sponsorship should be treated as formal, resourced components of leadership development, particularly for Māori leaders who may be isolated in predominantly non-Māori environments [21,44,49].

Organisational conditions that enable Māori leadership are also central. Where institutions do not change, hybrid leadership can become one-sided, with Māori leaders carrying disproportionate adaptation burdens [21,23]. Health services can respond by strengthening Māori authority in governance, embedding shared decision-making, protecting space for tikanga-based leadership practice, and developing equity accountability arrangements that are enforceable and resourced [15,57,58]. That health services champion these responses is critical given existing evidence that organisational barriers and limited Māori decision-making power remain persistent issues across all parts of the health system [16,57].

To make these recommendations concrete, four operational examples are offered. First, clear Māori decision rights mean establishing explicit governance provisions that give Māori board members, rūnanga, or advisory bodies binding rather than merely advisory authority in defined decision domains—for example, over service design for Māori populations, workforce equity targets, and equity accountability reporting. The distinction between binding authority and advisory input is critical: the latter places Māori knowledge within the system without conferring Māori authority. Second, resourced Māori-led priority setting means providing dedicated funding for iwi and Māori community organisations to lead their own needs assessments and participate in commissioning discussions from the outset, rather than being consulted only after funders have already set priorities. Third, embedding tikanga in how decisions are made means structuring governance meetings, service agreements, and accountability processes to include te reo, pōwhiri, and wānanga as standard practice rather than ceremonial addition, and building tikanga-based accountability into contracts. Fourth, making equity and anti-racism measurable requires health organisations to report against Māori-defined equity indicators with set targets and enforceable consequences, and to subject those reports to regular external Māori oversight rather than internal self-assessment alone.

The literature also aligns Māori leadership constraints with wider evidence on racism as a determinant that shapes Māori experiences within health systems and health workforces. Consequently, policy and practice responses must include measurable organisational accountability and resourcing for wholescale structural change, not simply individual-level, cultural competence initiatives [5,7,11]. Finally, protecting Māori leadership integrity within hybrid practice requires avoiding superficial “integration” of Māori values where power and decision rights remain unchanged. The evidence supports approaches that treat Māori leadership as legitimate governance practice and that build institutional arrangements that protect Māori authority and accountability relationships in health settings [15,25,58].

## 5. Conclusions

This review suggests that Māori health leadership is not simply a set of personal attributes that can be “added” to existing organisational models. It is a form of practice shaped by relationships, responsibilities, and institutional context. In health settings, leadership is often exercised at the boundary between iwi and community expectations and Crown-derived obligations, including within organisations whose structures and decision-rights have not been designed around Māori authority. This boundary work makes hybridity a common pragmatic requirement. Leaders are expected to interpret multiple accountabilities, translate priorities across different governance systems, and maintain integrity when institutional incentives pull in competing directions.

At the same time, the review highlights that hybrid leadership can become uneven when organisational change is limited. If systems treat Māori leadership primarily as representation, advice, or cultural performance, leaders can be left carrying a disproportionate burden of translation and relationship maintenance without the authority or resourcing needed to shift outcomes. These dynamics risk exhausting leaders, narrowing leadership pipelines, and may reproduce the very inequities that reforms are intended to address. For health services, the key implication is that strengthening Māori leadership is closely tied to redesigning the conditions in which leadership occurs. This may include clarifying Māori decision-rights in governance, resourcing Māori-led priority setting, and embedding accountability mechanisms that make equity and anti-racism operational responsibilities rather than aspirational statements.

The evidence base remains uneven across the health sector. Much of the available research is richly contextual, but there is limited comparable evidence across different levels of the health system, and limited examination of how particular leadership practices connect to changes in commissioning, service quality, and equity outcomes. Future work should therefore focus on the organisational mechanisms that enable Māori authority to be enacted in practice, including how leadership roles interact with accountability settings, funding and commissioning, and local partnership arrangements. It should also examine how leadership pathways are supported or constrained over time, including the pressures that fall unevenly on wāhine Māori and on leaders who are expected to hold cultural and institutional responsibilities simultaneously.

Overall, the review suggests that preparing future Māori health leaders requires more than individual capability development. It also requires institutional settings that can sustain Māori leadership with integrity, distribute leadership work collectively, and make system change achievable rather than dependent on exceptional individual effort.

## Data Availability

The original contributions presented in this study are included in the article/Supplementary Material. Further inquiries can be directed to the corresponding author.

## Supplementary Materials

Electronic searches across 12 bibliographic databases and two dedicated websites are provided in Supplementary Table S1, Supplementary S2, Supplementary S3 and Supplementary S4. Full search strings, database limits (Supplementary S4), and result counts are provided in Supplementary Table S5. The PRISMA 2020 checklist and ENTREQ statement are provided in Supplementary Table S6 and Supplementary Table S7. The concept matrix template and decision rules are reported in Supplementary S8 and Supplementary Table S9. Appraisal and data extraction details are in Supplementary Table S10. The following supporting information can be downloaded at: https://www.mdpi.com/article/10.3390/ijerph23050559/s1. References [62,63] are cited in the supplementary materials.

## Glossary

The following Indigenous Māori words are used in this manuscript:

Aotearoa	New Zealand (the Māori name for New Zealand)
ariki	paramount chief, supreme chief, high chief
aroha	love, compassion, empathy, sympathy, caring, kindness, deep affection, consideration, benevolence
hapū	kinship group, clan, sub-tribe—section of a large kinship group consisting of a number of whānau sharing descent from a common ancestor
hauora	health, wellbeing
hui	gathering, meeting, assembly, conference
hūmārietanga	modesty, humility, meekness
iwi	extended kinship group, tribe, nation, people, nationality, race
kaitiakitanga	guardianship, stewardship, trusteeship, care, protection (especially of the environment)
kaupapa	topic, subject, purpose, agenda, philosophy, strategy, plan
kaupapa Māori	Māori approach, Māori topic, Māori customary practice, Māori institution, Māori agenda, Māori principles, Māori ideology
kaumātua	elder, elderly person, old person, adult, elderly man or woman, respected elder
kawa	protocol, ritual, ceremonial system, ceremonies
kāwanatanga	government, governance, dominion, rule
Māori	indigenous New Zealander, indigenous person of Aotearoa/New Zealand, normal, usual, natural, fresh (of water)
mana	prestige, authority, control, power, influence, status, spiritual power, charisma
manaakitanga	hospitality, kindness, generosity, support, care, showing respect and kindness to others, hosting
Manatū Hauora	Ministry of Health
manawānui	perseverance, patience, steadfastness, endurance, determination
māia	courage, bravery, confident, brave
marae	courtyard, plaza, public forum, communal/sacred place that serves religious and social purposes
mātauranga Māori	Māori knowledge, wisdom, understanding, skill
Ngāi Tahu	South Island tribal confederation, major iwi of the South Island
Ngāti Porou	tribal region on the East Coast of the North Island
Pae Ora	Healthy Futures (health framework)
Pākehā	New Zealander of European descent, foreigner
pono	truth, integrity, truthfulness, honesty, sincerity, loyalty, faithfulness
pōwhiri	welcoming ceremony, welcome, invitation
pūrākau	narratives, stories, myths, legends
rangatahi	Young adult, youth, young person
rangatira	chief, leader, person of rank, chiefly person
rangatiratanga	chieftainship, leadership, sovereignty, self-determination, self-management, authority, ownership
rohe	ancestral territory, region, district, area
taiao	environment, natural world, nature
Tāngata whenua	Indigenous people; people of the land; Māori
tangihanga	funeral, rites for the dead, funeral ceremony
tautoko	support, prop, advocate for, encourage, back up
tika	correct, right, true, just, fair, accurate, appropriate, lawful, justice, correctness
tikanga	correct procedure, custom, habit, lore, method, manner, rule, way, code, meaning, plan, practice, convention, protocol
Te Aka Whai Ora	Māori Health Authority (now disestablished)
te ao Māori	The Māori world, Māori worldview, Māori perspective
te reo Māori	the Māori language
Te Tau Ihu	region in the northern South Island of New Zealand
Te Tiriti o Waitangi	Treaty partnership agreement between Māori chiefs and the British Crown (signed in 1840). Not to be confused with Treaty of Waitangi—refers to the version written in English.
Te Tiriti	abbreviated reference to Te Tiriti o Waitangi
toa	warrior, brave person, champion, expert, brave, courageous, daring
tohunga	priest, healer, expert, skilled person, specialist
toi	art, knowledge, creative expression
tuakana-teina	a teaching and learning approach based on the relationship between an older/more expert person (tuākana) and a younger/less expert person (tēina), where guidance, support, and shared learning are reciprocal, each learns from and teaches the other.
ūkaipō	nurturing place, place of origin, home, source of sustenance, mother’s womb, place where one was nourished
wahine toa	Women warriors, strong/brave women
wāhine	woman, women, female, wife
wānanga	deliberative forum, seminar, educational seminar, tribal knowledge, learning, learning session, place or institution of learning
whānau	extended family, family group, a familiar term of address to a number of people
whanaungatanga	relationship, kinship, sense of family connection, relationships between people through shared experiences and working together
whakapapa	genealogy, lineage, descent, cultural identity, to recite genealogy
whakataukī	proverb, significant saying, proverbial saying
whenua	land; placenta
