# Supplementary material for: Hybrid Leadership for Māori Health: A Systematic Review
Source: Int J Environ Res Public Health. 2026 Apr 26;23(5):559. doi: 10.3390/ijerph23050559 (PMC13206435; doi:10.3390/ijerph23050559)
Supplement: Supplementary file 1 [file ijerph-23-00559-s001.zip › ijerph-4192092-supplementary.pdf]

Supplementary Material

Supplementary Table S.1: Information sources and platform coverage.

Databases	Websites
APA PsychINFO	http://www.hauhake.auckland.ac.nz/
AUS/NZ Reference Centre	www.NZresearch.org.nz
CINAHL Plus	
Google Scholar	
Informit	
Proquest	
PubMed	
SAGE Journals	
ScienceDirect	
Scopus via Elsevier	
Springerlink	
Wiley Online Library	

Supplementary S.2: Rationale for data sources

Following the guidelines of PRISMA and ENTREQ [1,2,3,4] each of the databases and websites searched was chosen because of the subject areas they cover and their use in previous literature reviews investigating Māori health-related topics [5,6,7,8]. APA PsychINFO, CINAHL Plus, PubMed, and Scopus are commonly searched for health-related literature. AUS/NZ Reference Centre and Informit are sources of New Zealand literature. Proquest, Sage Journals, ScienceDirect, SpringerLink, and Wiley Online Library are all multidisciplinary databases. Google Scholar provides access to a large pool of formally published and grey literature [9], including citations not typically found elsewhere [10]. NZrearch.org.nz provides access to research from New Zealand universities, polytechnics, and research organisations, including doctoral, master, and undergraduate theses. He Hauhake Whakaaro (Te Kupenga Hauora Māori [Department of Māori health], University of Auckland) provides access to Kaupapa Māori literature (including grey literature).

Supplementary S.3: Search Terms

The search terms used included key terms from this literature review's question and synonyms brainstormed by the team (*see* Table S5). PubMed's Medical Subject Headings (MeSH) were also checked for potential search terms [11]. Boolean AND/OR operators and wildcard symbols (e.g., ? or #) were used where possible. Wildcard symbols were used for the word *Māori*, which is also sometimes spelt *Maori* or *Maaori*. With databases where wildcard symbols were unavailable, searches using each of the alternative spellings of Māori took place. Table S5 details the search strings used in each database and the search terms used in websites.

Supplementary S.4: Search Limits

The limits used were based on the eligibility criteria for the literature review (*see* Eligibility Criteria section in the Methods and Materials section) and what limits each database, website, or search engine had available. Table S5 includes the limits and the number of results achieved in each database and website.

Supplementary Table S.5: Database and Website Searches.

Date searched	Database/ Website/ Academic Journal	Search String/search terms	Limits	Search results
1.03.2022	PsychInfo	("Māori leadership" OR "Māori decision-making") AND ("health equity" OR health) AND (Model OR Framework)	No limits	2
1.03.2022	AUS/NZ Reference Centre	("Māori leadership" OR "Māori decision-making") AND ("health equity" OR health) AND (Model OR Framework)	**Publication Date:** 2000-2022<br>**Expanders:**<br>1. Apply related words<br>2. Also search within the full text of the articles<br>3. Apply equivalent subjects<br>**Source Types Included**<br>• Academic Journals (Domestic)<br>• Academic Journals (International)<br>**Source Types Excluded**<br>• Newspapers (Domestic)<br>• Magazines (International)<br>• Magazines (Domestic)<br>**Search modes** - Boolean/Phrase	12
1.03.2022	CINAHL Complete	("Māori leadership" OR "Māori decision-making") AND ("health equity" OR health) AND (Model OR Framework)	No limits	3
1.03.2022	Google Scholar	("Māori leadership" OR "Māori decision-making") AND ("health equity" OR health) AND (Model OR Framework)	**Publication Date:** 2000-2022	734
		("Maori leadership" OR "Maori decision-making") AND ("health equity" OR health) AND (Model OR Framework)	**Publication Date:** 2000-2022	368
		("Maaori leadership" OR "Maaori decision-making") AND ("health equity" OR health) AND (Model OR Framework)	**Publication Date:** 2000-2022	5
1.03.2022	Informit	("Māori leadership" OR "Māori decision-making") AND ("health equity" OR health) AND (Model OR Framework)	No limits	0
		("Maori leadership" OR "Maori decision-making") AND ("health equity" OR health) AND (Model OR Framework)	No limits	1
		("Maaori leadership" OR "Maaori decision-making") AND ("health equity" OR health) AND (Model OR Framework)	No limits	0
1.03.2022	PubMed	("Māori leadership" OR "Māori decision-making") AND ("health equity" OR health) AND (Model OR Framework)	**Publication Date:** 2000-2022<br>**Language:** English	88
		("Maori leadership" OR "Maori decision-making") AND ("health equity" OR health) AND (Model OR Framework)	**Publication Date:** 2000-2022<br>**Language:** English	88

Date searched	Database/ Website/ Academic Journal	Search String/search terms	Limits	Search results
		("Maaori leadership" OR "Maaori decision-making") AND ("health equity" OR health) AND (Model OR Framework)	No limits	0
1.03.2022	Sage Journals	("Māori leadership" OR "Māori decision-making") AND ("health equity" OR health) AND (Model OR Framework)	No limits	27
		("Maori leadership" OR "Maori decision-making") AND ("health equity" OR health) AND (Model OR Framework)	**Publication Date:** 2000-2022	38
		("Maaori leadership" OR "Maaori decision-making") AND ("health equity" OR health) AND (Model OR Framework)	No limits	0
1.03.2022	Science Direct	("Māori leadership" OR "Māori decision-making") AND ("health equity" OR health) AND (Model OR Framework)	**Publication Date:** 2000-2022	11
		("Maori leadership" OR "Maori decision-making") AND ("health equity" OR health) AND (Model OR Framework)	**Publication Date:** 2000-2022	12
1.03.2022	Science Direct	("Maaori leadership" OR "Maaori decision-making") AND ("health equity" OR health) AND (Model OR Framework)	**Publication Date:** 2000-2022	0
1.03.2022	Scopus	("Māori leadership" OR "Māori decision-making") AND ("health equity" OR health) AND (Model OR Framework)	No limits	4
		("Maori leadership" OR "Maori decision-making") AND ("health equity" OR health) AND (Model OR Framework)	No limits	4
		("Maaori leadership" OR "Maaori decision-making") AND ("health equity" OR health) AND (Model OR Framework)	No limits	0
1.03.2022	SpringerLink	("Māori leadership" OR "Māori decision-making") AND ("health equity" OR health) AND (Model OR Framework)	**Publication Date:** 2000-2022	39
		("Maori leadership" OR "Maori decision-making") AND ("health equity" OR health) AND (Model OR Framework)	**Publication Date:** 2000-2022	47
		("Maaori leadership" OR "Maaori decision-making") AND ("health equity" OR health) AND (Model OR Framework)	**Publication Date:** 2000-2022	1
1.03.2022	Wiley Online Library	("Māori leadership" OR "Māori decision-making") AND ("health equity" OR health) AND (Model OR Framework)	**Publication Date:** 2000-2021 (2022 not available)	37
		("Maori leadership" OR "Maori decision-making") AND ("health equity" OR health) AND (Model OR Framework)	**Publication Date:** 2000-2021 (2022 not available)	37
		("Maaori leadership" OR "Maaori decision-making") AND ("health equity" OR health) AND (Model OR Framework)	**Publication Date:** 2000-2021 (2022 not available)	0
1.03.2022	Proquest	("Māori leadership" OR "Māori decision-making") AND ("health equity" OR	**Publication Date:** 2000-2022 **Source Types Included**	327

Date searched	Database/ Website/ Academic Journal	Search String/search terms	Limits	Search results
		health) AND (Model OR Framework)	• Books<br>• Scholarly journals<br>• Reports<br>• Conference papers & proceedings,<br>• Dissertations and thesis<br>**Source Types Excluded**<br>• Newspapers<br>• Wire Feeds<br>• Historical Newspapers	
		("Maori leadership" OR "Maori decision-making") AND ("health equity" OR health) AND (Model OR Framework)	**Publication Date:** 2000-2022<br>**Source Types Included**<br>• Books<br>• Scholarly journals<br>• Reports<br>• Conference papers & proceedings,<br>• Dissertations and thesis<br>**Source Types Excluded**<br>• Newspapers<br>• Wire Feeds<br>• Historical Newspapers	345
		("Maaori leadership" OR "Maaori decision-making") AND ("health equity" OR health) AND (Model OR Framework)	No limits	2
16.02.2022	He Hauhake Whakaaro (website)	Leadership	No limits	3
		Decision-making	No limits	16
16.02.2022	NZresearch.org.nz (website)	"Māori leadership" AND health	No limits	11
		"Māori decision-making" AND health	No limits	78

Supplementary Table S.6: PRISMA Checklist

PRISMA checklist template from: [38]. https://doi.org/10.1136/bmj.n160

Section and Topic	Item #	Checklist item	Location where item is reported
TITLE			
Title	1	Identify the report as a systematic review.	Page 1.
ABSTRACT			
Abstract	2	See the PRISMA 2020 for Abstracts checklist.	Abstract section and original narrative abstract (S.11).
INTRODUCTION			
Rationale	3	Describe the rationale for the review in the context of existing knowledge.	Introduction.
Objectives	4	Provide an explicit statement of the objective(s) or question(s) the review addresses.	Introduction.
METHODS			
Eligibility criteria	5	Specify the inclusion and exclusion criteria for the review and how studies were grouped for the syntheses.	Eligibility Criteria section in the Materials and Methods section.
Information sources	6	Specify all databases, registers, websites, organisations, reference lists and other sources searched or consulted to identify studies. Specify the date when each source was last searched or consulted.	In the Materials and Methods section. See also Table S.1 Information Sources and Platform Coverage and Table S.5 Database searches.
Search strategy	7	Present the full search strategies for all databases, registers and websites, including any filters and limits used.	Screening and section in the Materials and Methods section. See also S.3 Search Terms and Table S.5 Database and Website Searches.
Selection process	8	Specify the methods used to decide whether a study met the inclusion criteria of the review, including how many reviewers screened each record and each report retrieved, whether they worked independently, and if applicable, details of automation tools used in the process.	Screening and Selection in the Materials and Methods section. See also Search Terms (S.3).
Data collection process	9	Specify the methods used to collect data from reports, including how many reviewers collected data from each report, whether they worked independently, any processes for obtaining or confirming data from study investigators, and if	Analysis and Synthesis section in the Materials and Methods section.

Section and Topic	Item #	Checklist item	Location where item is reported
		applicable, details of automation tools used in the process.	
Data items	10a	List and define all outcomes for which data were sought. Specify whether all results that were compatible with each outcome domain in each study were sought (e.g. for all measures, time points, analyses), and if not, the methods used to decide which results to collect.	Eligibility Criteria, Screening and Selection, Appraisal and Data Extraction, Analysis and Synthesis (Section 2.2 – 2.5) in the Materials and Methods section. See also Search Terms (S.3).
	10b	List and define all other variables for which data were sought (e.g. participant and intervention characteristics, funding sources). Describe any assumptions made about any missing or unclear information.	Appraisal and Data Extraction in the Materials and Methods section.
Study risk of bias assessment	11	Specify the methods used to assess risk of bias in the included studies, including details of the tool(s) used, how many reviewers assessed each study and whether they worked independently, and if applicable, details of automation tools used in the process.	Not applicable as meta-synthesis took place.
Effect measures	12	Specify for each outcome the effect measure(s) (e.g. risk ratio, mean difference) used in the synthesis or presentation of results.	Not applicable as meta-synthesis took place.
Synthesis methods	13a	Describe the processes used to decide which studies were eligible for each synthesis (e.g. tabulating the study intervention characteristics and comparing against the planned groups for each synthesis (item #5)).	Screening and Selection section as well as the Appraisal and Data Extraction section in the Materials and Methods section. See also Appraisal and Extraction Tools (Table S.10).
	13b	Describe any methods required to prepare the data for presentation or synthesis, such as handling of missing summary statistics, or data conversions.	Not applicable as meta-synthesis took place.
	13c	Describe any methods used to tabulate or visually display results of individual studies and syntheses.	Screening and Selection section, and the Appraisal and Data Extraction section in the Materials and Methods section. See also Selection Process (S.8).
	13d	Describe any methods used to	Analysis and Synthesis section in the

Section and Topic	Item #	Checklist item	Location where item is reported
		synthesize results and provide a rationale for the choice(s). If meta-analysis was performed, describe the model(s), method(s) to identify the presence and extent of statistical heterogeneity, and software package(s) used.	Materials and Methods section.
	13e	Describe any methods used to explore possible causes of heterogeneity among study results (e.g. subgroup analysis, meta-regression).	Not applicable as meta-synthesis took place.
	13f	Describe any sensitivity analyses conducted to assess robustness of the synthesized results.	Not applicable as meta-synthesis took place.
Reporting bias assessment	14	Describe any methods used to assess risk of bias due to missing results in a synthesis (arising from reporting biases).	Not applicable as meta-synthesis took place.
Certainty assessment	15	Describe any methods used to assess certainty (or confidence) in the body of evidence for an outcome.	Not applicable as meta-synthesis took place.
RESULTS			
Study selection	16a	Describe the results of the search and selection process, from the number of records identified in the search to the number of studies included in the review, ideally using a flow diagram.	Figure 1. Prisma 2020 Flow diagram.
	16b	Cite studies that might appear to meet the inclusion criteria, but which were excluded, and explain why they were excluded.	Not applicable.
Study characteristics	17	Cite each included study and present its characteristics.	Appraisal and Extraction Tools/Templates: Tabulated Search Results (Table S.10).
Risk of bias in studies	18	Present assessments of risk of bias for each included study.	Not applicable as meta-synthesis took place.
Results of individual studies	19	For all outcomes, present, for each study: (a) summary statistics for each group (where appropriate) and (b) an effect estimate and its precision (e.g. confidence/credible interval), ideally using structured tables or plots.	Not applicable as meta-synthesis took place.
Results of syntheses	20a	For each synthesis, briefly summarise the characteristics and	Not applicable as meta-synthesis took place.

Section and Topic	Item #	Checklist item	Location where item is reported
		risk of bias among contributing studies.	
	20b	Present results of all statistical syntheses conducted. If meta-analysis was done, present for each the summary estimate and its precision (e.g. confidence/credible interval) and measures of statistical heterogeneity. If comparing groups, describe the direction of the effect.	Not applicable as meta-synthesis took place.
	20c	Present results of all investigations of possible causes of heterogeneity among study results.	Not applicable as meta-synthesis took place.
	20d	Present results of all sensitivity analyses conducted to assess the robustness of the synthesized results.	Not applicable as meta-synthesis took place.
Reporting biases	21	Present assessments of risk of bias due to missing results (arising from reporting biases) for each synthesis assessed.	Not applicable as meta-synthesis took place.
Certainty of evidence	22	Present assessments of certainty (or confidence) in the body of evidence for each outcome assessed.	Not applicable as meta-synthesis took place.
DISCUSSION			
Discussion	23a	Provide a general interpretation of the results in the context of other evidence.	Discussion section.
	23b	Discuss any limitations of the evidence included in the review.	Limitations section in the Materials and Methods section.
	23c	Discuss any limitations of the review processes used.	Limitations section in the Materials and Methods section. See also Table S.9 and Table S.10
	23d	Discuss implications of the results for practice, policy, and future research.	Discussion section.
OTHER INFORMATION			
Registration and protocol	24a	Provide registration information for the review, including register name and registration number, or state that the review was not registered.	Not applicable.
	24b	Indicate where the review protocol can be accessed, or state that a protocol was not prepared.	Not applicable.
	24c	Describe and explain any amendments to information	Not applicable.

Section and Topic	Item #	Checklist item	Location where item is reported
		provided at registration or in the protocol.	
Support	25	Describe sources of financial or non-financial support for the review, and the role of the funders or sponsors in the review.	Funding section.
Competing interests	26	Declare any competing interests of review authors.	Conflict of Interests section.
Availability of data, code and other materials	27	Report which of the following are publicly available and where they can be found: template data collection forms; data extracted from included studies; data used for all analyses; analytic code; any other materials used in the review.	Not applicable

Supplementary Table S.7: ENTREQ Statement

ENTREQ statement template adapted from[29]. https://doi.org/10.1186/1471-2288-12-181

No	Item	Guide and description	Section
1	Aim	State the research question the synthesis addresses.	Materials and Methods section
2	Synthesis methodology	Identify the synthesis methodology or theoretical framework which underpins the synthesis, and describe the rationale for choice of methodology (e.g. meta-ethnography, thematic synthesis, critical interpretive synthesis, grounded theory synthesis, realist synthesis, meta-aggregation, meta-study, framework synthesis).	Materials and Methods section, particularly the beginning and the Analysis and Synthesis section.
3	Approach to searching	Indicate whether the search was pre-planned (comprehensive search strategies to seek all available studies) or iterative (to seek all available concepts until they theoretical saturation is achieved).	Information Sources and Platform Coverage (Table S.1)
4	Inclusion criteria	Specify the inclusion/exclusion criteria (e.g. in terms of population, language, year limits, type of publication, study type).	Eligibility Criteria section in the Materials and Methods section.
5	Data sources	Describe the information sources used (e.g. electronic databases (MEDLINE, EMBASE, CINAHL, psycINFO, Econlit),	Information Sources and Platform Coverage (see Table S.1) as well as Rationale for Data Sources (S. 2).

No	Item	Guide and description	Section
		grey literature databases (digital thesis, policy reports), relevant organisational websites, experts, information specialists, generic web searches (Google Scholar) hand searching, reference lists) and when the searches conducted; provide the rationale for using the data sources.	
6	Electronic Search strategy	Describe the literature search (e.g. provide electronic search strategies with population terms, clinical or health topic terms, experiential or social phenomena related terms, filters for qualitative research, and search limits).	Screening and section in the Materials and Methods section. See also Database and Website Searches (Table S.5).
7	Study screening methods	Describe the process of study screening and sifting (e.g. title, abstract and full text review, number of independent reviewers who screened studies).	Screening and Selection section, in the Materials and Methods section. See also Selection Process (S.8).
8	Study characteristics	Present the characteristics of the included studies (e.g. year of publication, country, population, number of participants, data collection, methodology, analysis, research questions).	Appraisal and Extraction Tools/Templates: Tabulated search results (Table S.10).
9	Study selection results	Identify the number of studies screened and provide reasons for study exclusion (e,g, for comprehensive searching, provide numbers of studies screened and reasons for exclusion indicated in a figure/flowchart; for iterative searching describe reasons for study exclusion and inclusion based on modifications to the research question and/or contribution to theory development).	Figure 1. Prisma 2020 Flow diagram.<br><br>Screening and Selection section as well as the Appraisal and Data Extraction section in the Materials and Methods section. See also Selection Process (S.8).
10	Rationale for appraisal	Describe the rationale and approach used to appraise the included studies or selected findings (e.g. assessment of conduct (validity and robustness), assessment of reporting (transparency), assessment of content and utility of the findings).	Appraisal and Data Extraction section in the Materials and Methods section.
11	Appraisal items	State the tools, frameworks and criteria used to appraise the studies or selected findings (e.g. Existing tools: CASP, QARI, COREQ, Mays and Pope [25]; reviewer developed tools; describe the domains	Appraisal and Data Extraction section in the Materials and Methods section.

No	Item	Guide and description	Section
		assessed: research team, study design, data analysis and interpretations, reporting).	
12	Appraisal process	Indicate whether the appraisal was conducted independently by more than one reviewer and if consensus was required.	Appraisal and Data Extraction section in the Materials and Methods section.
13	Appraisal results	Present results of the quality assessment and indicate which articles, if any, were weighted/excluded based on the assessment and give the rationale.	Appraisal and Data Extraction section in the Materials and Methods section.
14	Data extraction	Indicate which sections of the primary studies were analysed and how were the data extracted from the primary studies? (e.g. all text under the headings "results /conclusions" were extracted electronically and entered into a computer software).	Appraisal and Data Extraction section, as well as the Analysis and Synthesis section in the Materials and Methods section.
15	Software	State the computer software used, if any.	Zotero (version 5): Screening and Selection section, and the Appraisal and Data Extraction section in the Materials and Methods section. See also Selection Process (S.8).<br>Dedoose (version 9.0.17): Analysis and Synthesis<br>ChatGPT-5.2: Use of Generative Artificial Intelligence
16	Number of reviewers	Identify who was involved in coding and analysis.	Authors contributions section
17	Coding	Describe the process for coding of data (e.g. line by line coding to search for concepts).	Screening and Selection section, as well as the Analysis and Synthesis section in the Materials and Methods section.
18	Study comparison	Describe how comparisons were made within and across studies (e.g. subsequent studies were coded into preexisting concepts, and new concepts were created when deemed necessary).	Screening and Selection section, as well as the Analysis and Synthesis section in the Materials and Methods section.
19	Derivation of themes	Explain whether the process of deriving the themes or constructs was inductive or deductive.	Analysis and Synthesis section in the Materials and Methods section.
20	Quotations	Provide quotations from the primary studies to illustrate themes/constructs and	Due to the volume of themes found, the results section was initially

No	Item	Guide and description	Section
		identify whether the quotations were participant quotations of the author's interpretation.	primarily narrative summaries
21	Synthesis output	Present rich, compelling and useful results that go beyond a summary of the primary studies (e.g. new interpretation, models of evidence, conceptual models, analytical framework, development of a new theory or construct).	Findings section.

Supplementary S.8: Selection Process

Round One: The descriptive metadata of the database and website search results were imported into the reference management software Zotero. For Google Scholar and ProQuest, which had 1107 and 674 search results respectively, only the first 200 results per search string were imported. Once the search results were in Zotero, duplicates were identified and merged so that only one copy existed of each text. R.M then read all the titles, abstracts, or available summary information to assess whether any results met this review's inclusion criteria. Items considered eligible moved to a second round of screening, the full-text stage. When it was difficult to determine whether a text should be included or excluded, they were included in the second round rather than creating a third *maybe* category. For the items short-listed for the second round of screening, a concept matrix tracking the presence of information related to the key concepts for this project. This concept matrix was used to gain an initial understanding of the types of information being identified in the literature (See Table S.9).

Questions considered when the concept matrix was being constructed:

- Have any leadership models been presented? (yes/no)
- Has traditional or modern Māori leadership (or both) been discussed? (traditional/modern/both)
- If traditional leadership is being discussed, is it fit for the contemporary context? (yes/no)
- Has any of the work arisen from the Treaty of Waitangi claims process? (yes/no)

Round Two: Full-text versions for all items that passed the first round of screening were retrieved and divided among the team (R.M., B.M.-A. and A.B.) to read independently. Again, the texts were assessed against the inclusion and exclusion criteria. The team members also built on the concept matrix created in round one. Team members noted additional information such as participants, methodology, and methods. This information assisted the team in checking inclusion criteria and gaining a deeper understanding of the literature. The team discussed the texts and decided which would be included in the review.

Reasons for excluding texts during round two included:

- Not being focused on what Māori leadership is.
- Not health or iwi-focused.
- Not primary qualitative research.
- Focused on patient decision-making.

Supplementary Table S.9: Example of Concept Matrix

• = YES

Author (year)	leadership (L) or decision-making (D)	Health related	Traditional Leadership			Contemporary Leadership		
			Model	Underlying principles discussed*	Fit for contemporary context	Model	Underlying principles discussed*	Arisen from Treaty of Waitangi claims process
Example (2012)	L	•					•	
Example (2013)	L		•	•	•			
Example (2017)	L	•				•	•	
Example (2020)	L			•	•			•

*Includes: cultural values, skills, abilities, and processes utilised and valued by Māori or Indigenous people

Supplementary Table S.10: Appraisal and Extraction Tools/Templates: Tabulated Search Results.

Author (Year)	Aim	Location	Participants	Methodology	Data Collection	Analysis	Publication Type
Bean (2018)	Identify, describe, and elaborate on the critical concepts of Māori leadership	New Zealand	34 leaders from the public sector (national and regionally focused), 85.3% (29) were Māori.	Kaupapa Māori	Interviews, wānanga, and case study	Sense-making, narrative analysis, Kaupapa Māori analysis, and comparative analysis	Doctoral thesis
Palmer & Masters (2010)	Explores pathways for Māori women into and within sport leadership	New Zealand	Four Māori female sports leaders	Kaupapa Māori, Māori feminist, intersectionality theory	Semi-structured interviews	Cross-case analysis	Peer-reviewed journal article
Stevenson (2013)	To find out how Māori leadership is expressed in a recreation organisation and gain understanding of the experiences of particular Māori leaders	Ngāi Tahu region, New Zealand	Seven Māori leaders from a cultural and leadership development programme (employees and graduates)	Kaupapa Māori	Case study, face-to-face Interviews	Inductive analysis	Master's thesis
Taiapa (2019)	Explore the relationship between mana and leadership within a Māori	Ngāti Porou region, New Zealand	Four Māori and two Pākehā. All employees of a collectively owned hapū (sub-	Māori Centred Approach	Case study, one-one semi-structured interviews	Thematic analysis	Master's thesis

	organisation		tribe) organisation				
Te Rito (2007)	To identify the relationship that exists between Māori culture, rugby, and leadership	Auckland, New Zealand	Nine Māori male rugby leaders	Grounded Theory, Kaupapa Māori	Semi-structured interviews	Thematic analysis	Master's thesis
Tipene (2017)	To explore the experience and meaning of Māori leadership for Māori practitioners working in New Zealand public health units	New Zealand	11 Māori from public health units who held technical, clinical, management, or leadership positions	Critical Hermeneutic and Kaupapa Māori	Individual interviews	Hermeneutic interpretative approach and kaupapa Māori critique	Master's thesis
Tonumaipe'a (2018)	To study how Māori cultural values have assisted Māori business leaders who internationalise in China	New Zealand	Five Māori leaders in small business	Kaupapa Māori, integration of Grounded Theory and Case Study	Face-to-face interviews	Integration of Grounded Theory and Case Study	Master's thesis
Williams (2016)	To examine the nature of Māori leadership in Te Tau Ihu iwi in the late twentieth century.	Te Tau Ihu, New Zealand	Three Te Tahu Ihu leaders (all Māori)	Kaupapa Māori, Oral History	Semi-structured Interviews	Thematic analysis	Honours dissertation
Wirihana (2012)	This study sought to explore the life experiences of Māori women leaders and how these have been influential on their roles as leaders in Māori communities	New Zealand	Eleven Māori women leaders from a variety of sectors/disciplines (includes health)	Pūrākau	Interviews	Analysis framework based on Pūrakau	Doctoral thesis

Supplementary S.11: Original Narrative Abstract

This systematic review synthesises qualitative literature on Māori leadership to examine how leadership is conceptualised, enacted, and constrained, and what this implies for Aotearoa New Zealand's health system. Across included studies, Māori leadership is grounded in whakapapa-based legitimacy, tikanga and mātauranga Māori, and collective responsibility for relational, cultural, and intergenerational wellbeing; these foundations persist across "traditional" and "contemporary" settings, with differences reflecting institutional conditions rather than shifts in core values. Interpreting the literature through a Māori cultural lens, the review shows that leadership is often exercised within Crown-dominated organisations where Māori authority is not the default, requiring leaders to navigate multiple accountabilities to iwi and communities, organisational mandates, and statutory obligations. Hybridity emerges as a structurally produced feature of practice, integrating Māori relational ethics with bureaucratic, professional, and governance requirements and ongoing translation work to make Māori priorities legible within institutional systems. Health-sector evidence illustrates how commissioning, funding, and accountability arrangements can limit Māori decision-making, increase leadership burden, and constrain sustainability and leadership pipelines. The review concludes that strengthening Māori leadership in health requires organisational and system change—such as clearer Māori decision rights, resourced Māori-led priority setting, and accountability mechanisms that operationalise equity and anti-racism—alongside targeted research on governance, commissioning, and system design.

References

1. Page, M. J., McKenzie, J. E., Bossuyt, P. M., Boutron, I., Hoffmann, T. C., Mulrow, C. D., Shamseer, L., Tetzlaff, J. M., Akl, E. A., Brennan, S. E., Chou, R., Glanville, J., Grimshaw, J. M., Hróbjartsson, A., Lalu, M. M., Li, T., Loder, E. W., Mayo-Wilson, E., McDonald, S., … Moher, D. The PRISMA 2020 statement: An updated guideline for reporting systematic reviews. *BMJ* **2021**, 372, n71. https://doi.org/10.1136/bmj.n71
2. Tong, A., Flemming, K., McInnes, E., Oliver, S., & Craig, J. Enhancing transparency in reporting the synthesis B of qualitative research: ENTREQ. *BMC Med. Res. Methodol.* **2012**, *12*(1), 181. https://doi.org/10.1186/1471-2288-12-181
3. Petticrew, M., & Roberts, H. (2006). *Systematic Reviews in the Social Sciences: A Practical Guide*. Oxford: Blackwell Publishing. https://onlinelibrary.wiley.com/doi/book/10.1002/9780470754887 (accessed on 12 January 2026).
4. Bramer, W.M., Milic, J., & Mast, F. (2017). Reviewing retrieved references for inclusion in systematic reviews using Endnote. *J. Med. Libr. Assoc.* **2017**, *105*, 84 – 87. PMCID: PMC5234463 DOI: 10.5195/jmla.2017.111
5. Graham, R., & Masters-Awatere, B. Experiences of Māori of Aotearoa New Zealand's public health system: A systematic review of two decades of published qualitative research. Australian and *NZJPH* **2020**, *44*(3), 193-200. https://doi.org/10.1111/1753-6405.12971
6. Wilson, D., Moloney, E., Parr, J. M., Aspinall, C., & Slark, J. Creating an Indigenous Māori-centred model of relational health: A literature review of Māori models of health. *J. Clin. Nurs.* **2021**, https://doi.org/10.1111/jocn.15859
7. Rahiri, J.-L., Alexander, Z., Harwood, M., Koea, J., & Hill, A. G. Systematic review of disparities in surgical care for Māori in New Zealand. *ANZ Journal of Surgery*, **2018**, 88(7–8), 683–689. https://doi.org/10.1111/ans.14310
8. Palmer, S. C., Gray, H., Huria, T., Lacey, C., Beckert, L., & Pitama, S. G. Reported Māori consumer experiences of health systems and programs in qualitative research: A systematic review with meta-synthesis. *Int J for Equity in Health*, **2019**, 18(1), 163. https://doi.org/10.1186/s12939-019-1057-4

9. Haddaway, N. R., Collins, A. M., Coughlin, D., & Kirk, S. The role of Google Scholar in evidence reviews and its applicability to grey literature searching. *PLoS ONE* **2015**, *10*(9), e0138237.
10. Martín-Martín, A., Thelwall, M., Orduna-Malea, E., & Delgado López-Cózar, E. Google Scholar, Microsoft Academic, Scopus, Dimensions, Web of Science, and Open Citations' COCI: A multidisciplinary comparison of coverage via citations. *Scientometrics* **2021**, *126*(1), 871–906. https://doi.org/10.1007/s11192-020-03690-4
11. National Centre for Biotechnology Information. (2022). MeSH. https://www.ncbi.nlm.nih.gov/mesh (S2) (accessed on 29 January 2022)
